# Non-Invasive Bioluminescence Imaging of β-Cell Function in Obese-Hyperglycemic [*ob/ob*] Mice

**DOI:** 10.1371/journal.pone.0106693

**Published:** 2014-09-08

**Authors:** Manishkumar Patel, Alexa Gleason, Stacey O'Malley, Brett Connolly, Donna Suresch, John Virostko, Neil Phillips, Shu-An Lin, Tsing-Bau Chen, Michael Klimas, Richard J. Hargreaves, Cyrille Sur, David L. Williams, Alvin C. Powers, Bohumil Bednar

**Affiliations:** 1 Imaging Department, Merck Research Laboratories, Merck and Co., West Point, Pennsylvania, United States of America; 2 Vanderbilt University Institute of Imaging Science, Vanderbilt University, Nashville, Tennessee, United States of America; 3 Department of Molecular Physiology and Biophysics, Vanderbilt University, Nashville, Tennessee, United States of America; 4 Division of Diabetes, Endocrinology, and Metabolism, Department of Medicine, Vanderbilt University, Nashville, Tennessee, United States of America; 5 Veterans Affairs Tennessee Valley Healthcare System, Nashville, Tennessee, United States of America; Johns Hopkins University, United States of America

## Abstract

**Background:**

Type 2 diabetes results from failure of the β-cells to compensate for increased insulin demand due to abnormal levels of metabolic factors. The *ob/ob*(lep-/-) mouse has been extensively studied as an animal model of type 2 diabetes. Previous studies have shown a correlation between β-cell function and bioluminescent imaging in lean genetically engineered mice. The ability to noninvasively monitor β-cell function in *ob/ob* mice could provide new information on β-cell regulation in type 2 diabetes.

**Methods:**

To create the B6 Albino *ob/ob* MIP-luc mice (ob/ob-luc), the *ob/ob* mouse was crossed with the CD1 MIP-luc mouse. All mice were backcrossed over multiple generations to ensure the genetic background of the transgenic mice was over 96% similar to the background of the original *ob/ob* mouse. Animal weight, blood glucose levels, insulin in plasma, and in vivo bioluminescence (BLI) were monitored weekly or biweekly for up to 70 weeks of age. BL imaging was performed using IVIS Spectrum (Perkin Elmer) and calculated by integrating the bioluminescence signal between 5 and 10 min after i.v. injection of D-luciferin. Insulin immunohistochemistry determined islet beta cell count and insulin secretion assay determined islet insulin function.

**Results:**

There were significant increases in BLI and insulin levels as the *ob/ob*-luc mice aged while glucose levels gradually decreased. *Ob/ob*-luc were sacrificed at different time points to determine ex vivo BLI, islet function and total β-cell numbers using a cell counting training algorithm developed for the Vectra image analysis system (Perkin Elmer). The number of β-cells increased as the mice aged and all three ex vivo measurements correlated with BLI.

**Conclusions:**

The *ob/ob*-luc mice can serve as a model of metabolic stress, similar to human type 2 diabetes using BLI as a surrogate marker for β-cell function.

## Introduction

The failure of insulin-producing pancreatic β-cells in type 2 diabetic patients is a consequence of high metabolic stress. Glucotoxicity and lipotoxicity imposed on the β-cells initiates functional changes such as reduced insulin secretion, ATP production and pulsatility of insulin release with a concurrent increased proinsulin/insulin ratio and production of reactive oxygen and nitrogen species, eventually resulting in an increase in β-cell apoptosis [1]. However, the ex-vivo experiments with β-cells indicate that the functional changes are reversible [2]. Further progress in understanding molecular processes controlling development of type 2 diabetes and advancement in the development of new therapeutics would benefit from animal models allowing noninvasive detection of pancreatic β-cell function.

The *ob/ob* mouse model, deficient in leptin, and *db/db* mouse, with mutated leptin receptor, are well studied models of obesity and diabetes. The *ob/ob* mice are hyperphagic, hyperglycemic and hyperinsulinemic with reduced metabolic rate [3]. They are considered a model for the pre-diabetic state with moderate hyperglycemia, high adiposity and large pancreatic islets containing high amount of insulin-producing β-cells. Mice become overweight shortly after the birth and at the same time develop hyperinsulinemia [4]. They become hyperglycemic during the first four weeks of life and glucose levels rise to reach a maximum between 3–5 months of age while the insulin levels peak later in life. With the increased insulin production the glucose levels start to decrease and become nearly normal at old age although there is significant variability between animals [3]. However, the *ob/ob* mice remain insulin resistant throughout their life [3], [5], [6].

Bioluminescence imaging, a highly sensitive optical imaging technology [7], has been successfully applied for *in vivo* detection of insulin production in pancreatic β-cells in mice [8], [9], [10]. Experiments with transgenic animals expressing the optical reporter luciferase, under control of the mouse insulin promoter in CD-1 and FVB/NJ mouse strains, demonstrated quantitative detection of pancreatic islets *in vivo* [8], [11]. Bioluminescence detected as few as 50 islets and provided a sensitive way to longitudinally follow luciferase-expressing islets after transplantation into the liver of FVB mice [12], [13], [14]. Optical measurement of the insulin production is a sensitive technique for the detection of β-cell function in vivo and ex vivo, after islet isolation [14]. However, these studies were performed in lean mice artificially induced using high fat diets. The ability to noninvasively monitor β-cell mass and their function in *ob/ob* mice could provide new information on β-cell regulation in a metabolic stress model similar to human type 2 diabetes. In order to noninvasively monitor β-cell mass and function in *ob/ob* mice we crossed the *ob/ob* mouse with the CD1 MIP-luc mouse. In this manuscript we describe the development of an *ob/ob* mouse model expressing the optical reporter luciferase in β-cells of pancreatic islets. We evaluated this mouse model by measuring animal weight, blood glucose levels, insulin in plasma, and in vivo bioluminescence weekly or biweekly for up to seventy weeks of age and comparing them to control mice.

## Materials and Methods

### Ethics Statement

All mice were bred and housed at Taconic and all experiments were approved by the Merck Institutional Animal Care and Use Committee (IACUC).

### Mouse Lines

We created the B6 Albino MIP-luc *ob/ob* mice (*ob/ob*-luc) mouse by crossing CD-1MIP-luc mice (a kind gift from Dr. Graeme Bell (University of Chicago, IL)) and B6 Albino mice (provided by The Jackson Laboratory, Bar Harbor, Maine) to create B6 Albino MIP-luc mice using speed congenics. B6 *ob/ob* mice (provided by Taconic, Cranbury Township, New Jersey) were also crossed with B6 Albino mice to create B6 albino *ob/ob* mice. These B6 Albino MIP-luc (lean control) mice were crossed with B6 albino *ob/ob* mice to create the B6 Albino *ob/ob* MIP-luc mice (Figure S1). Both genders of mice homozygous for the B6 Albino and the *ob/ob* genes, and carriers of the MIP-luc genes were used for experimentation.

### In-vivo bioluminescent measurements

In-vivo bioluminescent measurements of both lean and *ob/ob*-luc mice were taken once every two weeks after they reached 7–8 weeks of age. The mice were fasted for 16 h prior to imaging, but were provided water ad libitum. For i.v. injection the mice were placed in a warming chamber and then injected with 150 µL of 15 mg/mL D-luciferin stock solution i.v. For i.p. injection the mice were injected at a dose of 72 mg/kg. The animals were weighed and then anesthetized with 3% isoflurane gas anesthesia. Five minutes after the D-luciferin injection, the animals were placed on the stage of the IVIS Spectrum (PerkinElmer, Waltham MA) with the animal's left side facing the CCD camera. Default bioluminescent settings of Living Image 4.0 were used with scanning times of five minutes with the F-stop adjusted to prevent image saturation. Regions of interest were placed on the 2D bioluminescent image to encompass the entire bioluminescent signal, and total photons emitted from the pancreas were calculated.

### Ex-vivo bioluminescent measurements

The pancreas was removed from both fasting and non-fasting mice for further evaluation. The pancreas was incubated in 0.15 mg/mL D-luciferin and imaged in the IVIS Spectrum (PerkinElmer, Waltham MA) immediately. Regions of interest (ROIs) were placed on the 2D bioluminescent image to encompass the entire pancreas and analysis was done using Living Image 4.0. The pancreas was then weighed and placed in formalin for histologic evaluation.

### Blood collection for glucose and insulin measurements

Blood was collected for glucose and insulin measurements post BLI. An AlphaTrak (Abbott) blood glucose monitoring system was used to measure glucose and insulin was measured using an AlphaLISA assay (Perkin Elmer) following manufacturer's protocol.

### Immunohistochemistry

After completing the ex vivo imaging each pancreas was immersed in 10% buffered formalin, fixed for 24–48 h, and routinely processed and embedded in paraffin. Four-micron thick sections were generated at 50-micron intervals through the paraffin block until the entire tissue block was exhausted, resulting in 11–20 separate levels depending upon the size of the pancreas. To identify pancreatic β-cells, one section from each 50-micron interval of each pancreas was immunostained using a murine reactive guinea pig anti-insulin antibody (ab7842; Abcam, Inc., Cambridge MA). For the entire study 635 slides were immunostained. Staining was performed on an automated immunostainer (intelliPATH FLX, BioCare Medical, Concord CA). Briefly, after removal of paraffin in xylene and hydrating the sections through graded ethanols, endogenous peroxidase was blocked by incubation in 3.0% H_2_O_2_. Non-specific binding sites were blocked by incubation with protein blocking solution (Sniper, BioCare) followed by incubation with the anti-insulin antibody (1∶50; 60 min RT). Sections were subsequently incubated with peroxidase-conjugated goat anti-guinea pig IgG F(ab')2 antibody (#106-036-003; 1∶1000; 30 min RT, Jackson ImmunoResearch Labs, Inc., West Grove PA) and 3,3′-diaminobenzidine (DAB) chromogen. Sections were counterstained with hematoxylin.

### Beta cell quantitation

To quantitate the DAB staining (β-cell counting) from each of the 635 immunostained slides we used a high-throughput multispectral analysis system (Vectra, PerkinElmer, Waltham MA) equipped with InForm and Nuance software. Briefly, the tissue sections were batch scanned for image acquisition. The software contains a learn-by-example interface whereby the user can draw a number of representative regions of morphological interest (i.e. islets) as well as morphological regions to be excluded from analysis (i.e. exocrine pancreas), Training sets using both regions were generated and the resulting tissue segmentation algorithm was validated for accuracy before being run on all sections. For β-cell segmentation, a spectral library was generated to identify the hematoxylin stained nuclei and the DAB stained cytoplasm (β-cells) within the islets. Training sets were again generated and reviewed to test the accuracy/specificity of the cell segmentation algorithm in identifying only DAB stained cells. After this, islet segmentation and cell segmentation classifiers were verified and saved, automated scanning at 20x captured high resolution sub-images of the islets and, using the nuclear and cytoplasmic spectral libraries, only islet cells with DAB stained cytoplasm were counted. The number of β-cells counted from each 50 µm level of each pancreas was then summed to give to total number of β-cells per mouse. In this study over 93,000 20x high resolution images were captured and analyzed to acquire the data set.

### Insulin Secretion Assay

Islets were isolated by dissection of the splenic portion of the pancreas and collagenase P digestion as previously described [15]. Islets were handpicked under microscopic guidance and washed three times with 10 mM phosphate-buffered saline (PBS) containing 1% mouse serum and suspended in 30 µL of the same solution. For insulin content measurements, isolated islets were homogenized in acid alcohol (1 ml 12 M HCL/110 mL 95% ethanol) and incubated for 48 h at 4°C under mild agitation. The homogenate was centrifuged at 2,500 rpm for 30 min at 4°C. The supernatant was collected for radioimmunoassay and stored at −20°C, as described [16].

A cell perifusion system was used to test the response of isolated islet preparations to stimuli of 16.7 mM glucose and 16.7 mM glucose +50 µM isobutyl methyl xanthine (IBMX), as described [17].

## Results

### Creation of B6 Albino *ob/ob* MIP-luc mice

**Figure 1:**
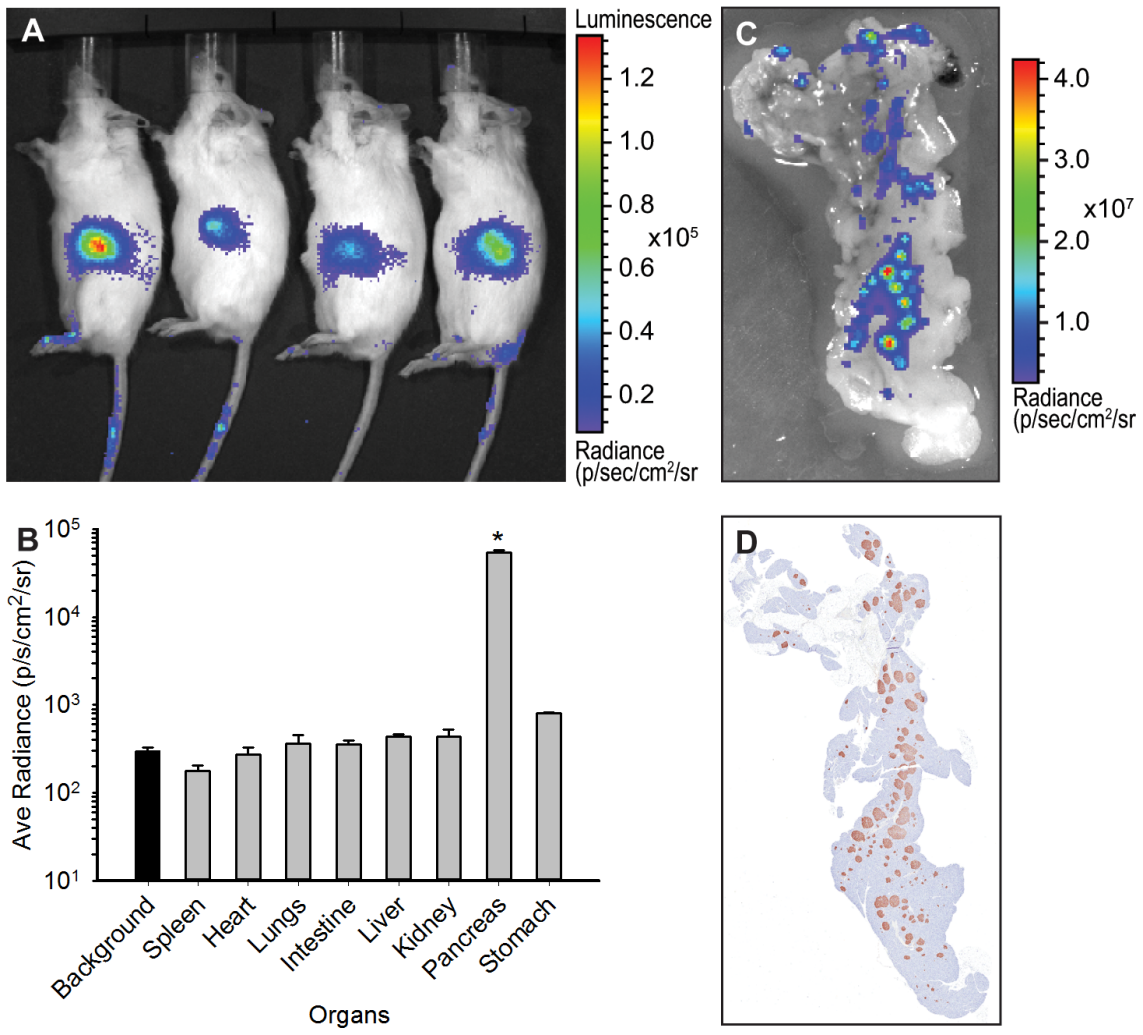
Luciferase expression is restricted to the pancreas. A) An example of BLI on *ob/ob*-luc mice. The animals of the same age were injected i.p. with D-luciferin and imaged 5 min later. Signal is present in the midsection of the animals only. B) Biodistribution of luciferase in the organs of *ob/ob*-luc mice. Three animals with similar intensities in the midsection were dissected and the organs were imaged with an IVIS Spectrum. ROI analysis was performed on the organs. The background bar is highlighted to show all organs except pancreas are at background levels while pancreas is significantly higher than other organs (*p<0.001, One way ANOVA). C) Zoomed in BLI of a pancreas. D) The pancreas was sectioned and stained with anti-insulin antibody. Both BLI and insulin staining have similar punctate patterns.

Figure S1 outlines the mating scheme used to create *ob/ob*-luc mice. MIP-Luc was introduced into B6 background by mating the CD1 MIP-Luc mice with B6 albino mice. Multiple backcrossing was used to ensure that the genetic background of the mice was <96% similar to B6 strain of mice. This was done due to the strain specificity of the ob/ob phenotype [18], [19] and allowed us to use this mouse as direct comparator, serving as the lean control. At the same time the albino phenotype was introduced into the ob mouse background to make the mice more amenable to BLI. This mating was used to create B6 albino ob/-. This mouse was mated to the lean mice and the F1 generation of this pairing was mated to each other to generate the *ob/ob*-luc mice. As the ob phenotype is infertile [20] when homozygous, the breeding pairs were heterozygous for the ob gene. This resulted in 25% of the mice being homozygous for ob gene and these mice were used throughout the study. Signal was present in the midsection of the animal (Figure 1a). Upon dissection, the bioluminescence was isolated to the pancreas only and not present in any other organs (Figure 1b), thus the expression pattern of the original MIP-Luc mouse [11] was maintained even with genetic manipulation. A close up of the pancreas reveals punctate bioluminescence that is similar to punctate anti-insulin staining of the histological sections of these pancreas (Figure 1c and d). This similar pattern of insulin staining and luciferase expression further confirmed that genetic manipulation of MIP-Luc mouse did not alter expression patterns of luciferase.

### Bioluminescent imaging (BLI) of *ob/ob*-luc mice requires i.v. injection of D-Luciferin

When the animals were first imaged, there was a high degree of variability between age-matched mice (Figure 1a). This could be due to animal to animal variation in the model itself or due to BLI procedure. Standard operating procedure for BLI calls for an intraperitoneal (i.p.) injection of D-luciferin, followed by a set absorption period and then imaging [21]. To determine if it is animal to animal variation or variation in the assay itself we performed a test-retest kinetic scans of the animals after i.p. and i.v. administration of D-luciferin. IP administration requires reabsorption from the peritoneal cavity to the bloodstream that can vary between and within animals. In addition the high amount of adipose tissue in these animals can cause differential kinetics of D-luciferin distribution leading to further inconsistent results as D-luciferin might be retained in the fat. D-luciferin that is administered i.v. has direct access to the blood stream and thus the tissues. Test-retest scans were performed every other day for three days on a total of 12 animals. Figure S2 shows examples of dynamic profiles of photon emission after i.p. (Figure S2a and b) and i.v. (Figure S2c and D) administration of D-luciferin. For i.p. administration, the time to peak and the peak itself is different among the days tested even within the same animal. Since the photon emission is instantaneous for i.v., administration, the time to peak is consistent as is the signal intensity. The area under the curve (AUC) was calculated for each day and averaged for each animal. This data was then used to calculate the coefficient of variation (CV) for each animal and then averaged across all animals for each delivery method. For i.p. administration the CV was 51.2% while that of i.v. administration was 8.3% (Table 1). To reduce the amount of time the animals are under anesthesia and to allow higher throughput of scanning, we decided to take one 5 min acquisition 5 min after i.v. administration of D-Luciferin. The CV for these measurements did not differ from the CV taken for the entire dynamic profile (Table 1).

**Table 1:** **Coefficient of Variation for Area Under the Curve measurements of i.p. vs. i.v. administration of D-luciferin in** ***ob/ob*** **-luc mice.**

	CV for Entire AUC (%)	CV for AUC from 5–10 min (%)
IP	51.2±22.3	57.2±26.7
IV	8.3±6.7	10.29±6.5

Test-retest scans were performed every other day for three days on a total of 12 animals. I.P. administration was tested the first week and i.v. administration was tested the following week and dynamic profiles were generated as described in Figure S2. AUC for the entire time course and for just 5–10 min interval was measured for each day and the Coefficient of Variation (CV)  =  ((STD/AUC)*100) was calculated for all three days for each mouse. The CV was then averaged for all 12 mice. I.V. is more consistent than i.p. and there is no statistical significant difference in performing a 5 min acquisition 5 min after D-luciferin administration vs. measuring for the entire time course.

### Longitudinal imaging of *ob/ob*-luc mice reveals that the bioluminescence increases with age

**Figure 2:**
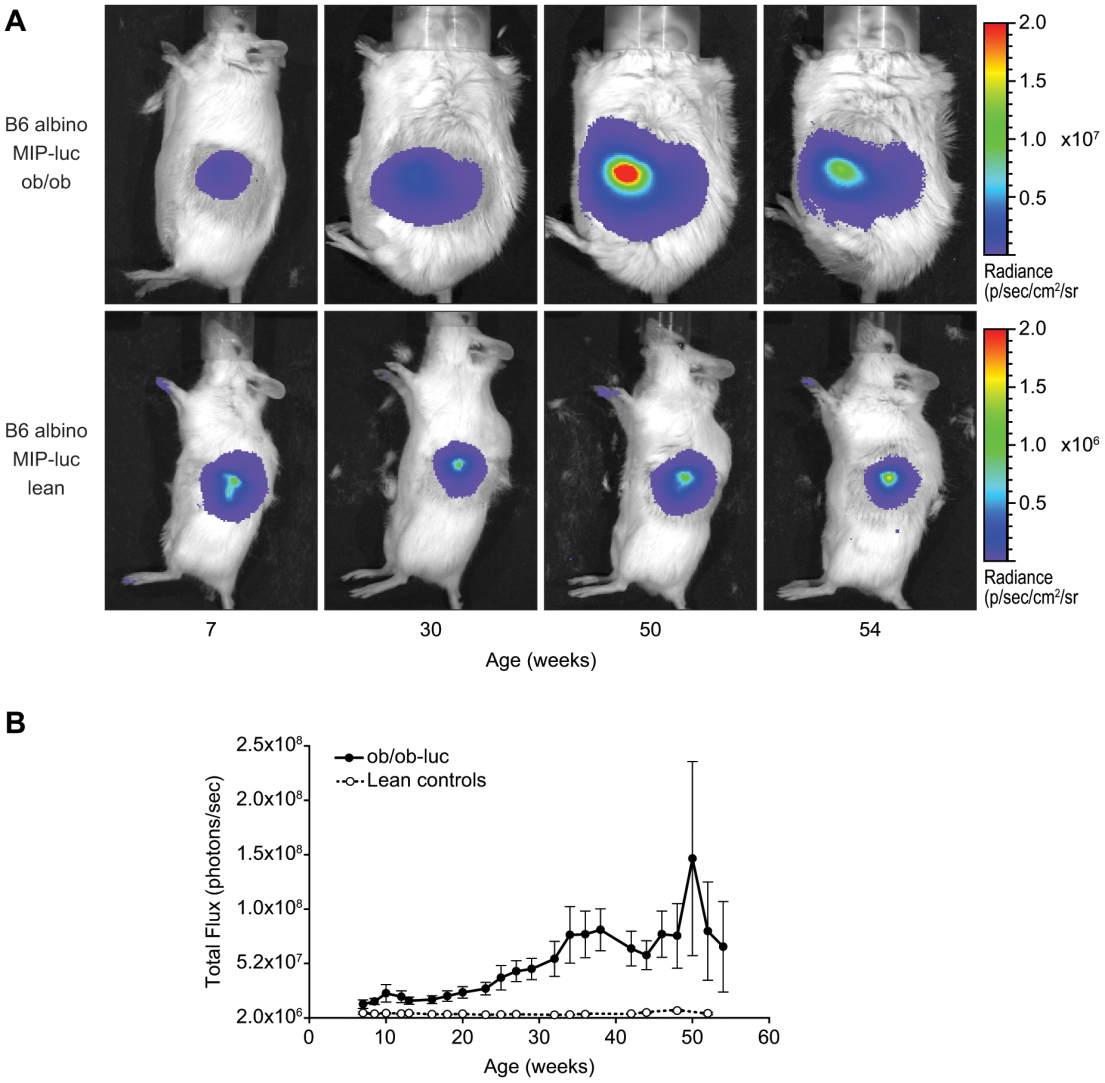
Longitudinal images of B6 albino MIP-luc lean (lean controls) and B6 albino MIP-luc *ob/ob* (*ob/ob*-luc) mice. A) Representative BLI of *ob/ob*-luc and lean controls over time. In *ob/ob*-luc mice the area of signal also increases, which could indicate an increase in the number of islets with age. However, the scatter of photons by adipose tissue may also contribute to the size of the detected area. Note: lean control mice are on a separate color scale since *ob/ob*-luc mice have higher luciferase expression B) Results of ROI analysis was performed on the pancreas signal of both *ob/ob*-luc (closed symbols) and lean control mice (open symbols) (N = 13 each). The ROI was drawn over the entire signal and presented as total flux (p/s). In *ob/ob*-luc mice the signal increases with age while in lean controls it remains unchanged.

Using the imaging procedure described above, we longitudinally followed the luciferase expression by BLI in *ob/ob*-luc mice and lean controls. The lean controls have the same genetic background as the *ob/ob*-luc mice except they are able to make leptin. Since the luciferase gene is driven by the mouse insulin promoter, and could be affected by food intake, the mice were fasted overnight prior to imaging and each imaging experiment took place at the same time of day. Figure 2a shows representative images of *ob/ob*-luc and control mice over time. ROI analysis of the signal showed an increase in bioluminescence with age in *ob/ob*-luc mice while the bioluminescence signal in the control mice remained constant (Figure 2b).

### Characterization of *ob/ob*-luc mice

**Figure 3:**
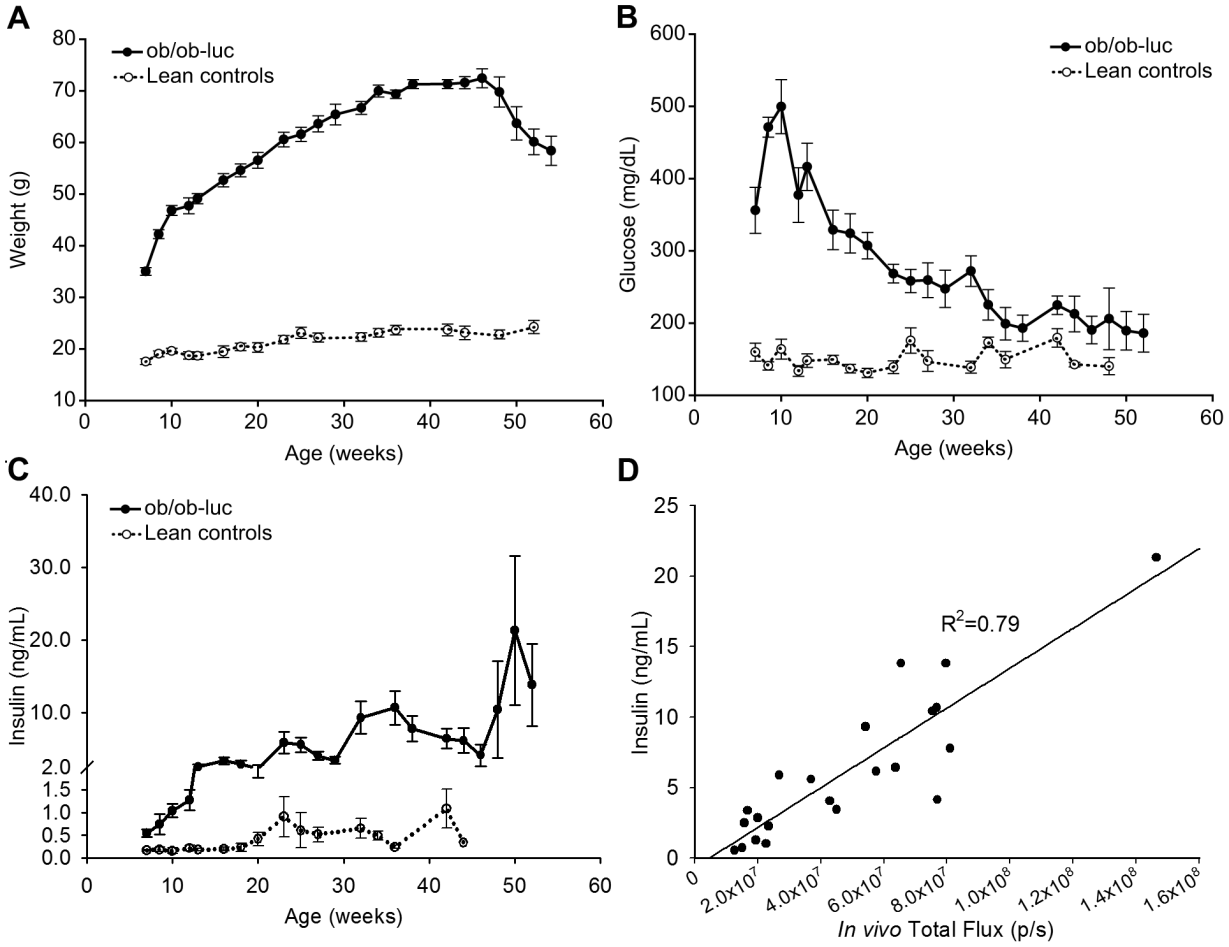
Characterization of *ob/ob*-luc mice. A) Weight increases significantly with age in *ob/ob*-luc mice consistent with the original *ob/ob* mouse model on the B6 black background. As early as week 8 the *ob/ob*-luc mice are 2.5 times the weight of their lean control counterparts. Blood was taken from fasting mice following imaging to measure B) glucose and C) insulin. Glucose levels in *ob/ob*-luc mice (closed symbols) are significantly higher than lean controls (open symbols) at week 8 (p<0.001, t-test). As the *ob/ob*-luc mice age the glucose levels decrease and reach similar levels as lean controls by week 45. Insulin levels in *ob/ob*-luc mice increase with age while insulin levels in lean mice remain stable with age. Both glucose and insulin levels follow similar trends as normal *ob/ob* mice, indicating that the additional genetic manipulation (albino and MIP-luc) did not affect the physical characteristics of the *ob/ob* model. D) There is a good correlation (R^2^ = 0.79) between plasma insulin levels and *in vivo* BLI measurements.

Physical characterization of the *ob/ob*-luc mice involved measurements of weight, insulin and blood glucose in both *ob/ob*-luc mice and lean controls. As expected, *ob/ob*-luc mice were significantly heavier than the lean control counterparts at 8 weeks of age, with *ob/ob*-luc mice weighing almost 2x more than lean controls (Figure 3a). *Ob/ob*-luc mice continued to gain weight with age, reaching 75–80 grams by 35 weeks of age. Glucose levels in *ob/ob*-luc mice were also significantly higher at 8 weeks compared to lean controls (p<0.001, t-test). Glucose levels in *ob/ob*-luc mice continued to increase until the mice reached 10 weeks of age (Figure 3b). In *ob/ob*-luc mice, insulin levels increased with age while insulin levels in lean mice remained constant (Figure 3c). As the insulin levels of *ob/ob*-luc mice increased (reaching a peak of 26.6±9.6 ng/ml at 34 weeks), glucose levels decreased, eventually reaching levels of lean controls (186±26 mg/dL at 52 weeks vs. 140±11 mg/dL). This pattern of glucose level changes in *ob/ob*-luc mice closely matches the glucose fluctuations observed in normal *ob/ob* mice [22]. Bioluminescence levels (Figure 2b) followed a similar trend to insulin levels and had a good correlation, R^2^ = 0.79 (Figure 3d).

### *Ex vivo* BLI correlates with the number of total β-cells in whole pancreas

**Figure 4:**
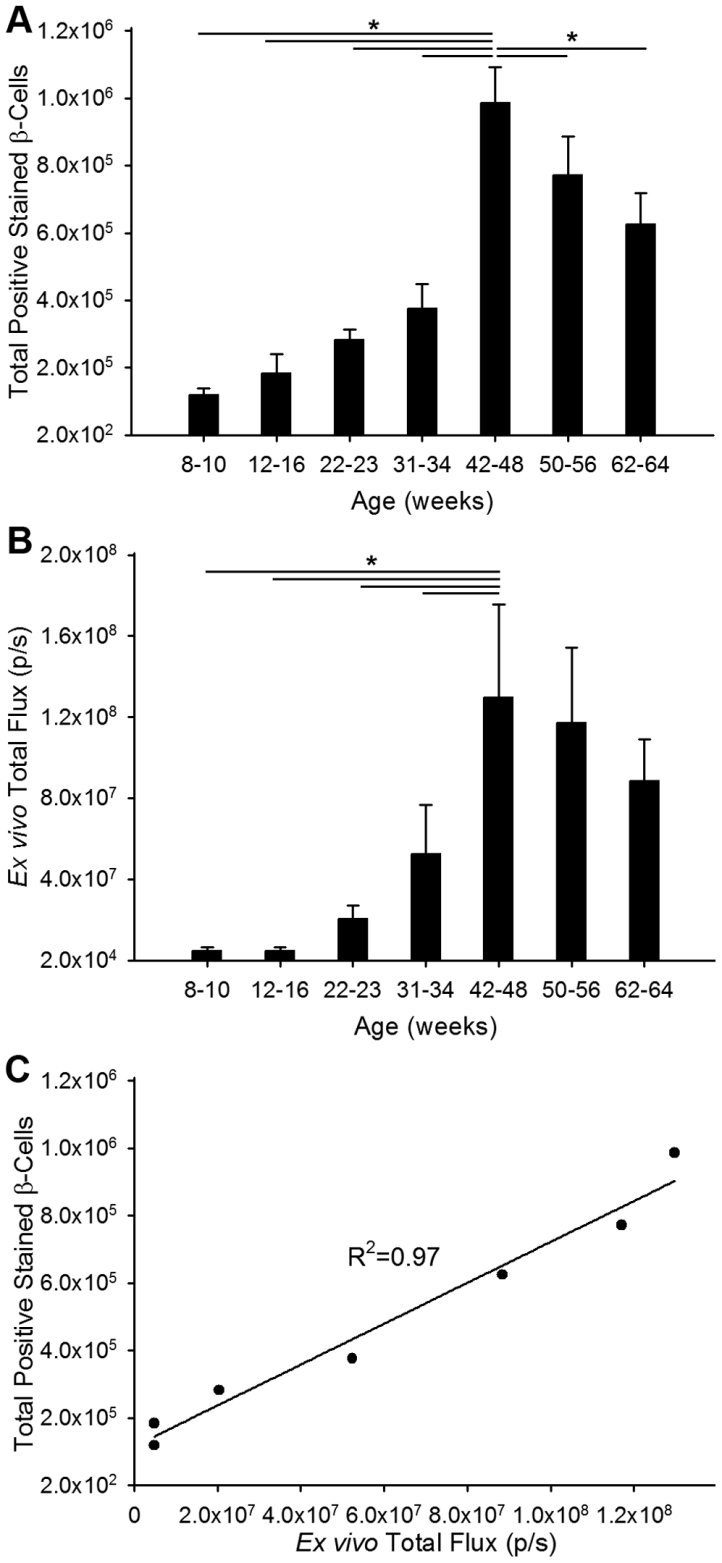
Correlation of *ex vivo* bioluminescence with quantitative histology. A) Total number of β-cells was counted from sections of pancreas from *ob/ob*-luc mice (N = 3–8/group). Mice were grouped to minimize variation since individual mice reach disease stages at different time points. B) Total bioluminescence was measured from *ex vivo* images of pancreata from *ob/ob*-luc mice. When the data are grouped similar to β-cell number there is an upwards trend in β-cell number as the mice age. (The horizontal lines in figures A and B indicate a statistically significant difference between the two groups, *p<0.001, One way ANOVA) C) There is a strong correlation between the number of β-cells counted by histology and *ex vivo* BLI measurements.

The pancreas of early, middle, and late aged *ob/ob*-luc mice were removed and prepared for histology as described in the methods section in order to correlate the number of β-cells to BLI. Therefore, to quantitate total β-cell number the entire pancreas was sectioned at 50 micron intervals then stained and each level was analyzed in a high-throughput multispectral analysis system (Vectra) that allows for automated counting of stained cells. This system works by training the software via a learn-by-example interface allowing the user to select regions of morphologic interest for analysis. We first trained the software to distinguish islets from all other elements on the slide (Figure S3a and b). Further training directed the software to identify islet cells that were positive in the insulin immunohistochemistry assays (Figure S3c, d, and e). The accuracy of these trainings was verified by examining random islet images after running the algorithms. Cell counting throughout the whole pancreas using the Vectra analysis system gives a more accurate depiction of the total β-cell number in each mouse than could be accomplished by manual counting. Also manual counting from a large number of slides is not practical. As the mice aged, the number of β-cells increased up to 48 weeks of age with a significant increase between ages 30 weeks and 40 weeks (p<0.001, ANOVA) (Figure 4a). Actual numbers are shown in Table S1. In contrast, the total number of β-cells in lean mice does not have the same dramatic increase with age (Table S1). Total bioluminescence, as measured in the pancreas *ex vivo*, also increased with age up to 48 weeks (Figure 4b). When the animals are grouped by age there is a high correlation between the number of β-cells and BLI (Figure 4c). After week 48, there is a statistically non-significant reduction in both the number of β-cells and bioluminescence as measured *ex vivo*. Non-uniform insulin staining with increasing age was also observed, which could represent the overall efficiency of the pancreas (Figure S4).

### *In vivo* BLI measurements correlate with *ex vivo* insulin measurements obtained directly from islet cells

**Figure 5:**
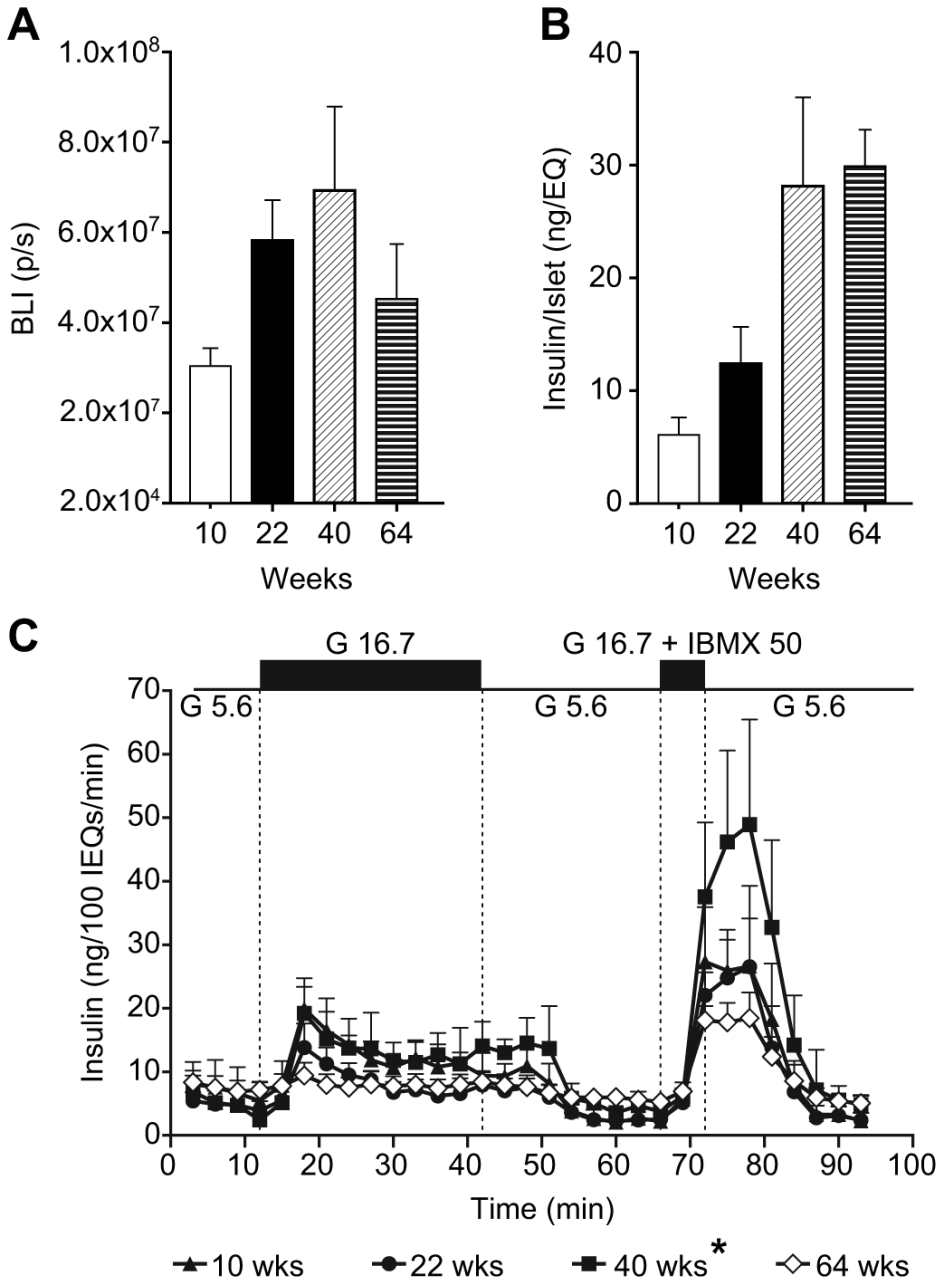
Insulin measurements on individual islets confirms *in vivo* BLI and insulin measurements. A) Bioluminescence imaging of islets isolated from *ob/ob* luciferase mice at 10, 22, 40, and 64 weeks of age displayed increased bioluminescence with increasing age up to 40 weeks of age, followed by decreased BLI at 64 weeks of age. B) Isolated islets displayed increased total insulin content with increasing age up to 40 weeks of age. Insulin content of islets from 64 week old mice was not significantly different than at 40 weeks. C) Isolated *ob/ob*-luc islets displayed insulin release in response to insulin secretagogues. The response from 64 week old islets is represented by open symbols to distinguish it from the other groups. High glucose (16.7 mM) and IBMX induced higher insulin release in islets from 40 week old mice and lower insulin release in islets from 64 week old mice (*p = 0.015, One way ANOVA).

To directly correlate BLI measurements during age progression we isolated islets from 10, 22, 40 and 64 week old mice and measured BLI, insulin content and insulin release directly from islets (Figure 5). In each group the number of islets remained the same however the islets were of differing size due to the increase in islet size as the mice age. As the mice aged there was an increase in BLI and insulin output per islet up to age 40 weeks. At age 64 weeks there was a reduction in BLI (Figure 5a) and even though this reduction was not statistically significant this downward trend matches the downward trend observed in *in vivo* measurements. There was a statistically significant reduction (p = 0.015) in insulin release in the islets (Figure 5c) from 64 week old animals when compared to 40 week old animals. This again correlates with the *in vivo* measurements and suggests that in vivo measurements are a true depiction of the biological process occurring in the animal. However in 64 week old animals, there was no significant change in insulin content (Figure 5b) compared to 40 week old animals. Due to the short half-life of luciferase, BLI will reflect only new insulin production, rather than insulin storage for long periods. Therefore the data indicates the overall pool of insulin in the β-cells stays the same, but the rates of new insulin production and release are reduced in older animals and this change can be determined non-invasively through BLI using the *ob/ob*-luc mouse model.

## Discussion

Creating a model to measure β-cell function is important to understanding and treating type 2 diabetes. To this end we crossed the MIP-luc mouse with the *ob/ob* mouse to follow β-cell function using BLI. Due to multiple crossings, it was important to characterize the model to ensure the ob/ob phenotype remained consistent with the original *ob/ob* mouse. We followed the mice for at least 50 weeks and measured blood glucose, insulin, weight, and bioluminescence. We further characterized the mice by correlating BLI with the number of β-cells as the mice aged in a systematic approach.

As this model is intended to be used in drug discovery, we first optimized BLI so that we could image the mice on an industrial scale. Aged matched mice had a high degree of variability when using the standard operating procedure of i.p. administration of D-luciferin. These mice have high amounts of adipose tissue in the abdominal area which leads to inconsistent D-luciferin delivery to the i.p. cavity. Therefore we performed i.v. administration of D-luciferin throughout the study to minimize assay variability. Test retest scans performed using i.v. dosing had significantly less standard deviation then i.p. dosing of D-luciferin in *ob/ob*-luc mice. Substrate availability is instantaneous after i.v. administration resulting in higher blood and tissue concentrations. This results in higher signal intensities after i.v. administration than i.p. administration and the rapid decay of the signal is a result of rapid elimination of the substrate from blood [23]. This rapid decay in signal requires immediate image acquisition after i.v. administration. To make acquisition less strenuous on the operator and to reduce the amount of time the mice are under anesthesia, we decided to perform one 5 min acquisition 5 min after i.v. dosing. Doing so did not compromise the final data and allowed us to scan more mice in a given day than if we had collected data for the entire signal decay. There was no statistically significant difference in the CV for 5 min acquisitions vs. the CV calculated over the entire time course. Therefore we were confident that the short image acquisitions would not lead to further assay variability.

At 8 weeks of age the body weight of the *ob/ob*-luc mice was significantly higher than lean controls. The body weight continued to increase as the mice aged up to 34–46 weeks. After 50 weeks of age, the weight decreased followed by death of the mice at around 60–70 weeks. Glucose levels were also significantly higher in *ob/ob*-luc mice compared to lean controls as early as 8 weeks of age. Between 10 to 12 weeks of age, the glucose levels started to decline but did not reach lean control levels until 50 weeks of age. This was consistent with glucose characteristics of original *ob/ob* mice [22], [24]. Insulin levels rose with age up to 50 weeks and then decreased. This decline coincided with a significant loss in weight and bioluminescence. Bioluminescence measurements followed a similar trend as insulin measurements and had a reasonable correlation (R^2^ = 0.79). Both *in vivo* BLI and plasma insulin levels were variable in mice older than 50 weeks and this likely reflects the variability in disease progression of individual animals and their response to metabolic stress. Grouping the animals into 4 to 6 week age groups helped decrease the variability and lead to a high degree of correlation between BLI and β-cell number (R^2^ = 0.97).

Previous studies have reported an increase in the number of β-cells with increasing glucose and insulin requirement in rats [25]. In *ob/ob*-luc mice, there was an increase in insulin requirement as the mice aged. As a result we observed a similar increase in the number of β-cells as the mice aged. To get an accurate quantitation of the number of β-cells, a systematic and automated method was used to count β-cells from multiple sections that encompassed the entire pancreas from each mouse. Previous studies using an exhaustive method to manually count β-cells in *ob/ob* pancreas found that islet volume increased and islet number did not change when comparing to lean controls [26]. However, that study only compared pancreas of 8 week old mice. In this study, islet volume was higher in *ob/ob*-luc mice than lean control and as the *ob/ob*-luc mice aged the islet number increased as well. We also observed less intense insulin staining in pancreata from older mice. When we accessed the islets from older mice on their ability to release insulin, we found the islets from older *ob/ob*-luc mice secreted more insulin than younger mice before decreasing at 64 weeks of age. In this assay, the same number of islets was used between age groups however the size of the islets was not controlled due to the large variation in islet size among *ob/ob*-luc islets. One explanation could be that even though these islets are less efficient at making insulin (as indicated by weak insulin staining) the increased size of the islets compensates and allows the pancreas as a whole to make more insulin. This increase in insulin correlated well with the increase in bioluminescence as the *ob/ob*-luc mice aged, which suggests that luciferase expression can be used as a surrogate marker for insulin production in these mice. By 50 weeks of age the number of β-cells began to decrease concomitant with a decline in bioluminescence. Decreased islet volume/mass is also a characteristic of type 2 diabetes patients when compared to healthy patients [27], [28], [29]. Interestingly, there was also a clear reduction in the amount of insulin released from islets harvested from 64 week old mice even though the total insulin content in these islets did not change. This is an indication that the islets are losing the ability to secrete insulin efficiently, a condition also seen in islets recovered from type 2 diabetic patients [27]. Our β-cell counting analysis showed a decrease in the number of β-cells at this age using insulin staining as the β-cell indication method. However this method does not quantitate the amount of insulin in the cells. β-cells with low amounts of insulin are counted the same as β-cells with high amounts of insulin. Therefore it was not possible in this context to count the number of islets with low insulin staining and those with high insulin staining. Doing so might indicate the overall health and function of the pancreas and reflect observations made by BLI. So even though total insulin content in the islets may not change its ability to secrete and synthesize new insulin does change and this change is reflected in the BLI measurements in *ob/ob*-luc mice.

## Conclusions

In this longitudinal study, we created a B6 Albino MIP-luc ob/ob mouse model to non-invasively assess β-cell function using quantitative bioluminescent imaging. By applying insulin-specific immunohistochemistry, we validated changes in the BLI signal by directly measuring the number of β-cells present in the pancreas of these mice over time. Moreover, glucose and insulin measurements indicated that the genetic manipulations (albino and MIP-luc) we employed to create this model did not affect the characteristics of the B6 ob/ob model. Taken together the *ob/ob*-luc mouse can serve as a model of metabolic stress on β-cells that is similar to human type 2 diabetes.

## Supporting Information

Figure S1 **Breeding scheme used to create B6 Albino MIP-luc** ***ob/ob*** **mice.**
(TIF)

Figure S2 **Representative dynamic profiles of the photon emission after IV and IP administration of D-luciferin.** A and B) Photon emission after i.p. administration of D-luciferin in two representative *ob/ob*-luc mice. Each animal was imaged three times on alternating days. 1 min acquisitions were performed every 2 min for 40 min. For each mouse a different peak signal was observed on each day as well as different signal peak times. On day 3 the substrate might have been trapped in the fat for mouse 4. C and D) Photon emission after i.v. administration of D-luciferin in the same mice as above. The same animal was injected i.v. three consecutive days and imaged 1 min after injection using 1 min acquisitions every 2 min for 30 min. The kinetics are more consistent for i.v administration than i.p. administration of D-luciferin. The area under the curve (AUC) was calculated for each animal over the entire imaging session and the coefficient of variation (CV) is shown for each graph. Mouse 4 had the worst CV of the i.v. group. To make imaging of *ob/ob*-luc mice less strenuous on the mice and the operator, one 5 min image acquisition was performed 5 min after i.v. administration as indicated by the arrows (Figure S2C).
(TIF)

Figure S3 **Quantitation of insulin staining.** A) Representative low magnification image of insulin staining in islet. B) InForm tissue segmentation separates islets (red) from other tissue/non-tissue elements (green). C) Representative 20x image of insulin staining. D) InForm nuclear segmentation identifies cell nuclei (green) within islets. E) InForm cytoplasmic segmentation identifies cells with DAB staining (β-cells, pseudocolor).
(TIF)

Figure S4 **Non-uniform insulin staining in older ob/ob-luc islets.** Representative islets after insulin staining from 50 and 64 week old ob/ob-luc mice. At 64 weeks when both β-cell number and BLI have decreased there is non-uniform insulin staining in the islets.
(TIF)

Table S1 **Average β-cell numbers as counted from whole pancreas of** ***ob/ob*** **-luc and lean mice and plotted in** **Figure 4** **.**
(DOCX)
