# Supplementary figures and images for: Non-Invasive Bioluminescence Imaging of β-Cell Function in Obese-Hyperglycemic [ob/ob] Mice
Source: PLoS One. 2014 Sep 8;9(9):e106693. doi: 10.1371/journal.pone.0106693 (PMC4157804; doi:10.1371/journal.pone.0106693)

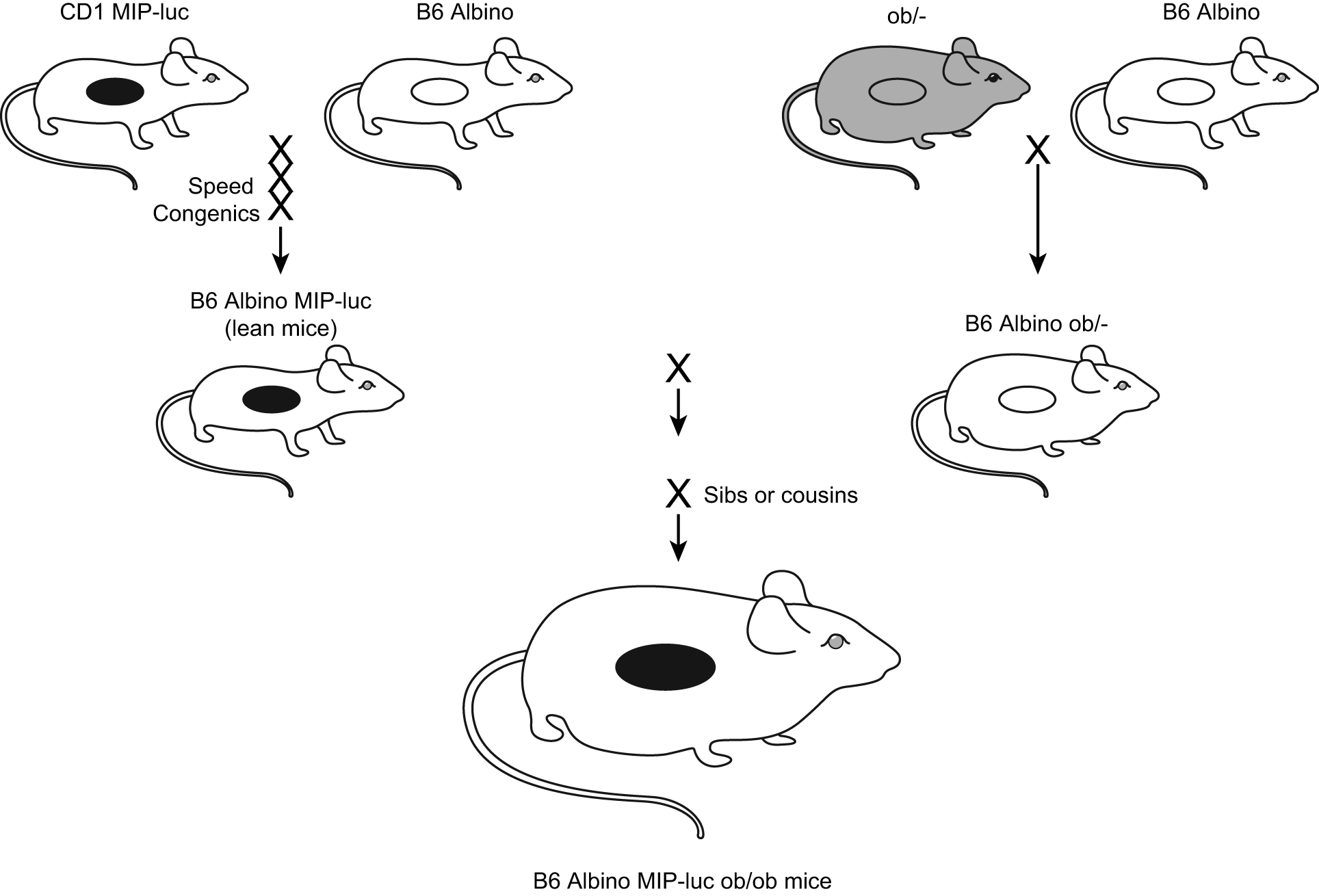

Supplement: Figure S1 — Breeding scheme used to create B6 Albino MIP-luc ob/ob mice. (TIF) [file pone.0106693.s001.tif]

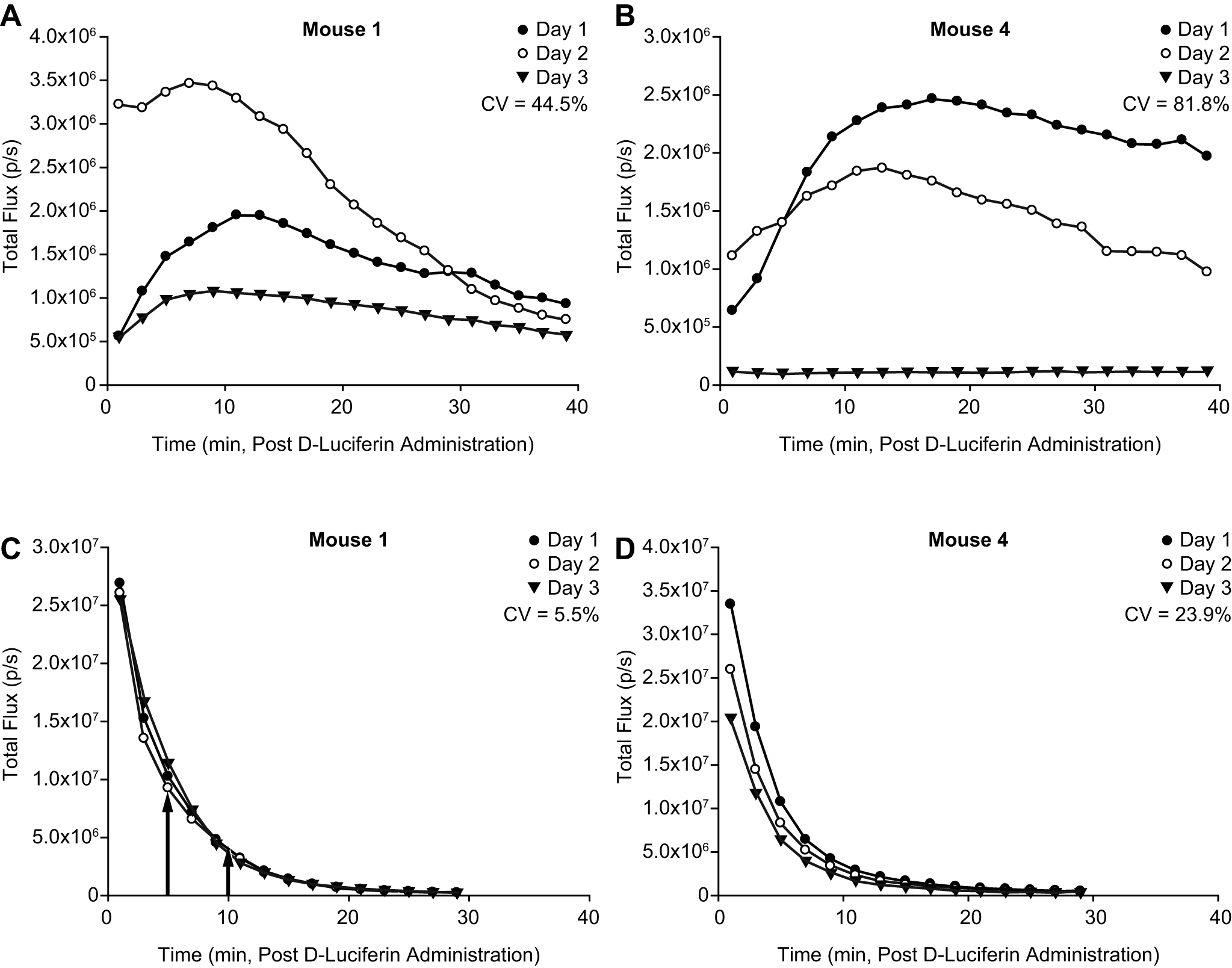

Supplement: Figure S2 — Representative dynamic profiles of the photon emission after IV and IP administration of D-luciferin. A and B) Photon emission after i.p. administration of D-luciferin in two representative ob/ob-luc mice. Each animal was imaged three times on alternating days. 1 min acquisitions were performed every 2 min for 40 min. For each mouse a different peak signal was observed on each day as well as different signal peak times. On day 3 the substrate might have been trapped in the fat for mouse 4. C and D) Photon emission after i.v. administration of D-luciferin in the same mice as above. The same animal was injected i.v. three consecutive days and imaged 1 min after injection using 1 min acquisitions every 2 min for 30 min. The kinetics are more consistent for i.v administration than i.p. administration of D-luciferin. The area under the curve (AUC) was calculated for each animal over the entire imaging session and the coefficient of variation (CV) is shown for each graph. Mouse 4 had the worst CV of the i.v. group. To make imaging of ob/ob-luc mice less strenuous on the mice and the operator, one 5 min image acquisition was performed 5 min after i.v. administration as indicated by the arrows (Figure S2C). (TIF) [file pone.0106693.s002.tif]

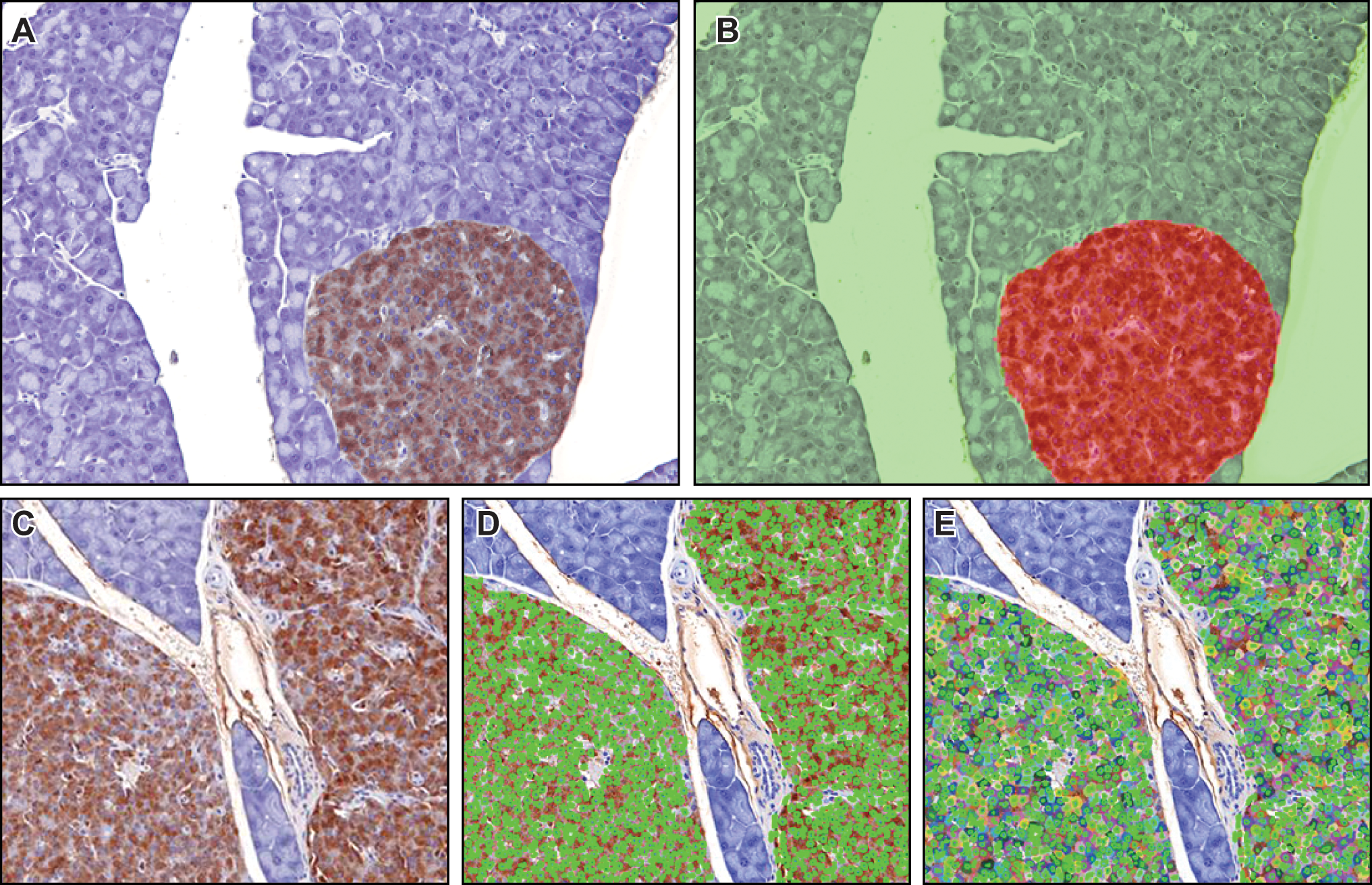

Supplement: Figure S3 — Quantitation of insulin staining. A) Representative low magnification image of insulin staining in islet. B) InForm tissue segmentation separates islets (red) from other tissue/non-tissue elements (green). C) Representative 20x image of insulin staining. D) InForm nuclear segmentation identifies cell nuclei (green) within islets. E) InForm cytoplasmic segmentation identifies cells with DAB staining (β-cells, pseudocolor). (TIF) [file pone.0106693.s003.tif]

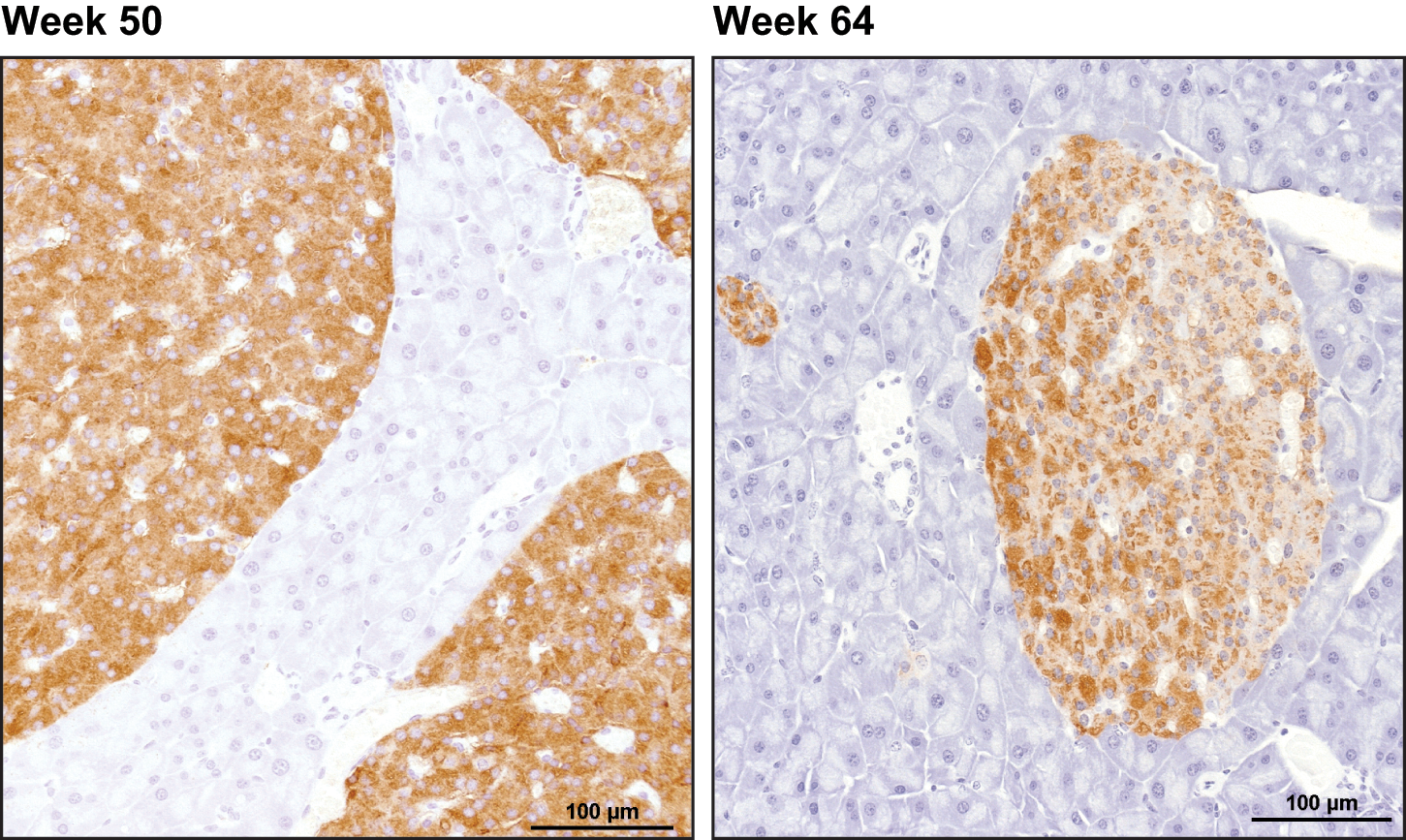

Supplement: Figure S4 — Non-uniform insulin staining in older ob/ob-luc islets. Representative islets after insulin staining from 50 and 64 week old ob/ob-luc mice. At 64 weeks when both β-cell number and BLI have decreased there is non-uniform insulin staining in the islets. (TIF) [file pone.0106693.s004.tif]
