# Supplementary material for: Non-Invasive Bioluminescence Imaging of β-Cell Function in Obese-Hyperglycemic [ob/ob] Mice
Source: PLoS One. 2014 Sep 8;9(9):e106693. doi: 10.1371/journal.pone.0106693 (PMC4157804; doi:10.1371/journal.pone.0106693)
Supplement: Table S1 — Average β-cell numbers as counted from whole pancreas of ob/ob -luc and lean mice and plotted in Figure 4 . (DOCX) [file pone.0106693.s005.docx]

| Age (Weeks) | *ob/ob*-luc | Lean |
| --- | --- | --- |
| 8-10 | 119060 ± 19382 | 11706 |
| 12-16 | 184391 ± 57149 |  |
| 22-23 | 283050 ± 31018 | 20576 |
| 31-34 | 376637 ± 71662 | 25487 |
| 42-48 | 986529 ± 105734 | 41596 ± 1890 |
| 50-56 | 771590 ± 114507 | 36935 ± 11727 |
| 62-64 | 624532 ± 93899 |  |
